# Structural diversity of neuronal calcium sensor proteins and insights for activation of retinal guanylyl cyclase by GCAP1

**DOI:** 10.3389/fnmol.2014.00019

**Published:** 2014-03-17

**Authors:** Sunghyuk Lim, Alexander M. Dizhoor, James B. Ames

**Affiliations:** ^1^Department of Chemistry, University of California at DavisDavis, CA, USA; ^2^Basic Sciences, Pennsylvania College of Optometry, Salus UniversityElkins Park, PA, USA

**Keywords:** calcium, EF-hand, Ca^2^^+^-myristoyl switch, NCS-1, recoverin, GCAP1, NCS protein, NMR

## Abstract

Neuronal calcium sensor (NCS) proteins, a sub-branch of the calmodulin superfamily, are expressed in the brain and retina where they transduce calcium signals and are genetically linked to degenerative diseases. The amino acid sequences of NCS proteins are highly conserved but their physiological functions are quite different. Retinal recoverin controls Ca^2^^+^-dependent inactivation of light-excited rhodopsin during phototransduction, guanylyl cyclase activating proteins 1 and 2 (GCAP1 and GCAP2) promote Ca^2^^+^-dependent activation of retinal guanylyl cyclases, and neuronal frequenin (NCS-1) modulates synaptic activity and neuronal secretion. Here we review the molecular structures of myristoylated forms of NCS-1, recoverin, and GCAP1 that all look very different, suggesting that the attached myristoyl group helps to refold these highly homologous proteins into different three-dimensional folds. Ca^2^^+^-binding to both recoverin and NCS-1 cause large protein conformational changes that ejects the covalently attached myristoyl group into the solvent exterior and promotes membrane targeting (Ca^2^^+^-myristoyl switch). The GCAP proteins undergo much smaller Ca^2^^+^-induced conformational changes and do not possess a Ca^2^^+^-myristoyl switch. Recent structures of GCAP1 in both its activator and Ca^2^^+^-bound inhibitory states will be discussed to understand structural determinants that control their Ca^2^^+^-dependent activation of retinal guanylyl cyclases.

## INTRODUCTION

**FIGURE 1:**
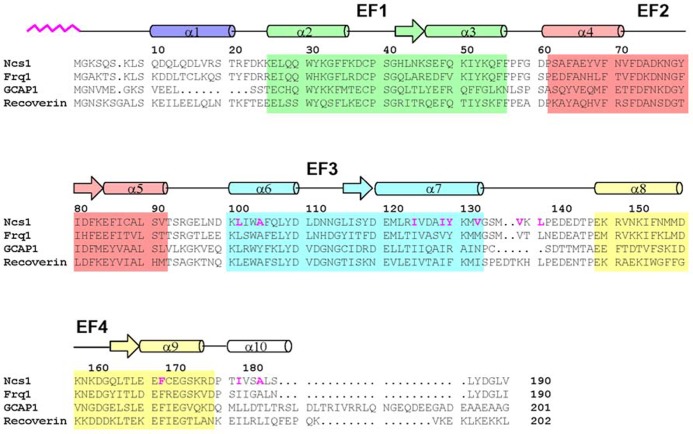
**Amino acid sequence alignment of selected NCS proteins (sequence numbering is for *S. * pombe NCS-1).** Secondary structure elements (helices and strands), EF-hand motifs (EF1 green, EF2 red, EF3 cyan, and EF4 yellow), and residues that interact with the myristoyl group (highlighted magenta) are indicated. Swiss Protein Database accession numbers are Q09711 (*S. pombe* NCS-1), Q06389 (*S. cerevisiae* Frq1), P21457 (bovine recoverin), and P43080 (human GCAP1).

Intracellular calcium ions (Ca^2^^+^) regulate neuronal signaling in the central nervous system (Berridge et al., 2000; Augustine et al., 2003). Neuronal Ca^2^^+^ signals are detected by a family of neuronal calcium sensor (NCS) proteins (Ames et al., 1996, 2012; Braunewell and Gundelfinger, 1999; Burgoyne and Weiss, 2001; Burgoyne et al., 2004; Weiss et al., 2010) that contain EF-hand motifs (Moncrief et al., 1990; Ikura, 1996; Ikura and Ames, 2006) as well as by a family of C_2_-domain containing proteins (synaptotagmin and protein kinase C isoforms; Nalefski and Falke, 1996; Corbalan and Gomez, 2014). At least sixteen different NCS proteins are known (Weiss and Burgoyne, 2002; Burgoyne and Haynes, 2012) and are conserved from yeast to humans (**Figure 1**). Recoverin (Dizhoor et al., 1991) and guanylyl cyclase activating proteins 1 and 2 (GCAP1 and GCAP2; Dizhoor et al., 1994; Palczewski et al., 1994) are expressed in the retina, where they regulate phototransduction in photoreceptor cells (Palczewski et al., 2000; Ames and Ikura, 2002; Stephen et al., 2008; Ames et al., 2012). NCS proteins are also expressed in the brain such as neurocalcin (Hidaka and Okazaki, 1993), frequenin (NCS-1; Pongs et al., 1993; McFerran et al., 1998), visinin-like proteins (VILIPs; Bernstein et al., 1999; Braunewell and Klein-Szanto, 2009), K^+^ channel interacting proteins (KChIPs; An et al., 2000), calsenilin/DREAM (Buxbaum et al., 1998; Carrion et al., 1999), and hippocalcin (Kobayashi et al., 1992, 1993; Tzingounis et al., 2007).

Recoverin also called S-modulin (Dizhoor et al., 1991; Kawamura and Murakami, 1991), the first NCS protein to be discovered, controls the lifetime of photo-excited rhodopsin (Kawamura, 1993; Erickson et al., 1998; Makino et al., 2004) by regulating rhodopsin kinase (Calvert et al., 1995; Chen et al., 1995; Klenchin et al., 1995; Komolov et al., 2009). Recoverin decreases the lifetime of rhodopsin at low Ca^2^^+^ levels to control visual recovery and promote photoreceptor adaptation to background light. More recent evidence indicates that recoverin can also modulate the decay of the light-activated phsophodiesterase activity. Such modulation may help accelerate visual recovery in the presence of background light (Chen et al., 2012). Recoverin is also located in the rod inner segment (Strissel et al., 2005) and is associated with cancer-associated retinopathy (Polans et al., 1991; Subramanian and Polans, 2004).

Guanylyl cyclase activating proteins 1 and 2 are also expressed in photoreceptor cells where they activate retinal guanylyl cyclase at low cytosolic Ca^2^^+^ levels upon light activation (Dizhoor et al., 1994; Palczewski et al., 1994, 2004). The EF-hand motifs in GCAPs can bind both Mg^2^^+^ and Ca^2^^+^ (Peshenko and Dizhoor, 2004, 2006). Mg^2^^+^ binding stabilizes a structural form of GCAPs that activates cyclase activity (Peshenko and Dizhoor, 2006; Lim et al., 2009), whereas Ca^2^^+^-bound GCAPs inhibit the cyclase (Dizhoor and Hurley, 1996; Dizhoor et al., 1998). GCAPs are important for regulating the recovery phase of visual excitation and particular mutants are linked to various forms of retinal degeneration (Semple-Rowland et al., 1996; Sokal et al., 1998; Baehr and Palczewski, 2007; Bondarenko et al., 2010; Jiang and Baehr, 2010).

Neuronal calcium sensor proteins (frequenin or NCS-1) are expressed in other tissues beside the brain (Kapp et al., 2003) and in lower organisms including flies (Pongs et al., 1993), worms (Gomez et al., 2001), and yeast (Frq1; Hendricks et al., 1999; Huttner et al., 2003; Hamasaki et al., 2004). Yeast NCS homologs (called Frq1) activate a phosphatidyl inositol 4-OH kinase isoform (Pik1; Hendricks et al., 1999; Kapp et al., 2003; Strahl et al., 2003, 2007) required for vesicle trafficking and secretion (Hama et al., 1999; Walch-Solimena and Novick, 1999). Mammalian NCS-1 interacts with voltage-gated Ca^2^^+^ and K^+^ channels (Weiss et al., 2000; Nakamura et al., 2001) and activates inositol trisphosphate receptors (Boehmerle et al., 2006).

NCS proteins typically contain about 200 amino acid residues in chain length with four EF-hand motifs, a first EF-hand that does not bind Ca^2^^+^, and a myristoylation consensus sequence at the N-terminus. NCS proteins have similar sequences, ranging from 35 to 60% identity (**Figure 1**). EF-hand residues are the most highly conserved, particularly in the Ca^2^^+^ binding loops. The fourth EF-hand sequence is variable, and Ca^2^^+^ is able to bind to EF4 in frequenin (Cox et al., 1994; Ames et al., 2000) and GCAPs (Peshenko and Dizhoor, 2007; Stephen et al., 2007) but Ca^2^^+^ does not bind to EF4 in recoverin (Ames et al., 1995a) and VILIPs (Cox et al., 1994; Li et al., 2011). Ca^2^^+^-binding to EF4 in GCAP1 controls whether GCAP1 can activate or inhibit guanylyl cyclase (Peshenko and Dizhoor, 2007). The residues near the C-terminus and linker between EF3 and EF4 are non-conserved, suggesting that these regions may play a role in target specificity for recoverin but not for GCAPs.

Retinal recoverin and most other NCS proteins are myristoylated at the amino terminus (Dizhoor et al., 1992; Kobayashi et al., 1993; Ladant, 1995). Recoverin and GCAPs contain a saturated myristoyl (14:0) or related fatty acyl group (12:0, 14:1, 14:2), because N-myristoyl transferase (Gordon et al., 1991) can efficiently utilize C12 and/or C14 acetyl-CoA as fatty acyl donors in the retina. In tissues other than the retina, myristoylation is the predominant modification. Myristoylated recoverin binds to cell membranes only at high Ca^2^^+^ levels (Zozulya and Stryer, 1992; Dizhoor et al., 1993), whereas unmyristoylated recoverin does not bind to membranes. Likewise, bovine neurocalcin (Ladant, 1995) and hippocalcin (Kobayashi et al., 1993) both are myristoylated and exhibit Ca^2^^+^-induced localization at the plasma membrane in response to neuronal stimulation. Ca^2^^+^-induced membrane targeting by NCS proteins has been termed, Ca^2^^+^-myristoyl switch. The attached fatty acyl group is buried inside the protein structure of Ca^2^^+^-free recoverin (Tanaka et al., 1995). Ca^2^^+^ binding to recoverin causes extrusion of the fatty acid, enabling it to interact with lipid bilayer membranes. Recoverin’s Ca^2^^+^-myristoyl switch may control its light-induced movement into the rod inner segment (Strissel et al., 2005). GCAP proteins are also myristoylated (Palczewski et al., 1994; Frins et al., 1996; Olshevskaya et al., 1997). However, unlike recoverin, GCAPs do not possess a functional Ca^2^^+^-myristoyl switch (Olshevskaya et al., 1997; Hwang and Koch, 2002). Instead the N-terminal myristoyl group remains sequestered inside GCAP1 in both Ca^2^^+^-free and Ca^2^^+^-bound states (Hughes et al., 1998; Lim et al., 2009). Indeed, the crystal structure of Ca^2^^+^-bound GCAP1 shows the myristoyl group surrounded by the protein (Stephen et al., 2007), and a recent nuclear magnetic resonance (NMR) structural analysis reveals that the activator state of GCAP1 has an overall structure similar to that of Ca^2^^+^-bound inhibitory state in which the N-terminal myristoyl group is buried in both the Ca^2^^+^-free and Ca^2^^+^-bound states (Lim et al., 2013).

**FIGURE 2:**
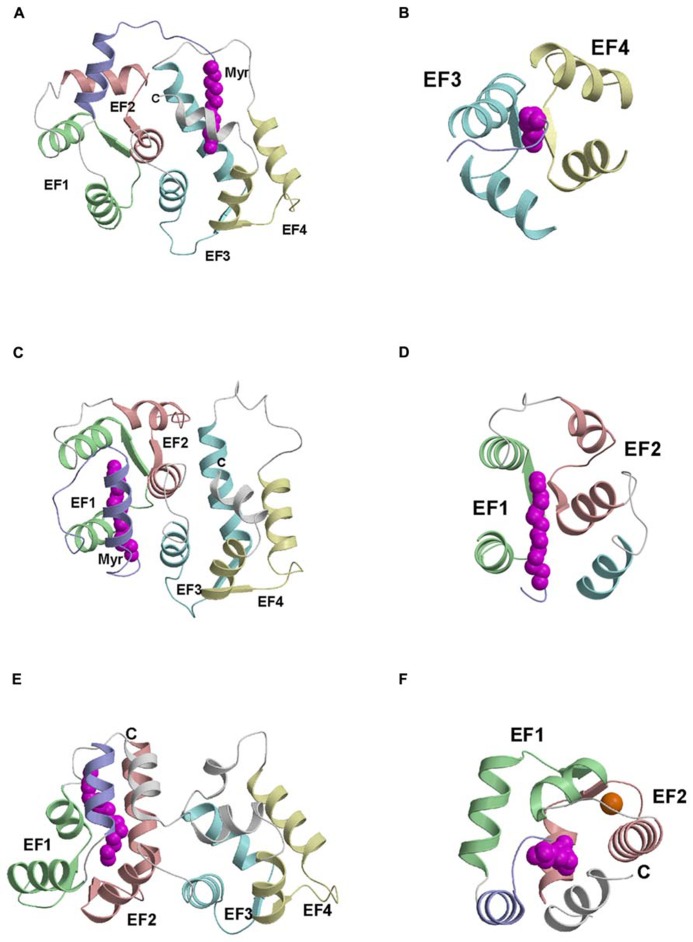
**Main chain structures of Ca^2+^-free myrisoylated NCS-1 (PDB ID: 212e) (A), recoverin (PDB ID: 1iku) (C), and GCAP1 (PDB ID: 2r2i) (E)**. Close-up views of the myristate binding pocket in NCS-1 **(B)**, recoverin **(D)** and GCAP1 **(F)**. EF-hands and myristoyl group (magenta) are colored as defined in **Figure 1**. Adapted from and originally published by Lim et al. (2011).

Atomic-resolution structures are known for myristoylated forms of recoverin (Ames et al., 1997), GCAP1 (Stephen et al., 2007), and NCS-1 (Lim et al., 2011) that each fold differently around the attached myristoyl group (**Figure 2**). For NCS-1, the attached myristoyl group is located in a protein crevice formed by helices from EF3 and EF4 near the C-terminus (**Figure 2A**). The covalently attached fatty acyl group in NCS-1 protrudes in a parallel fashion between four helices from EF3 and EF4 (**Figure 2B**). The C-terminal location of the myristoyl binding site in NCS-1 is quite different from that of recoverin in which the myristate is positioned inside a cavity near the N-terminus (**Figure 2C**). The attached fatty acyl chain in recoverin is wedged between the helices of EF1 and EF2 in a perpendicular fashion (**Figure 2D**), which contrasts with the parallel arrangement of the fatty acyl chain in NCS-1 (**Figure 2B**). For GCAP1, the myristoyl group is sequestered in a cavity formed by the N-terminal domain with participation of a C-terminal helix (**Figure 2E**). The myristoyl group in GCAP1 bridges the N-terminal and C-terminal ends of the protein by contacting helices at each end (**Figure 2F**). In short, the protein structural environment around the myristoyl group is quite different in recoverin, GCAP1 and NCS-1 (**Figure 2**). This suggests that each NCS protein folds differently around the N-terminal myristoyl group by contacting non-conserved patches of hydrophobic residues that are unique to each NCS protein. However, myristoylation of GCAP2 is not essential for its ability to activate its target (retinal guanylyl cyclase), because unmyristoylated GCAP2 can activate cyclase activity nearly as well as myristoylated GCAP2 (Olshevskaya et al., 1997).

In this review, we discuss recent structures of GCAP1 in both Ca^2^^+^-free activator and Ca^2^^+^-bound inhibitor states to understand structural determinants that control Ca^2^^+^-dependent activation of retinal guanylyl cyclases.

## Ca^2+^-DEPENDENT ACTIVATION OF RETINAL GUANYLYL CYCLASE

### GUANYLYL CYCLASE ACTIVATION AND PHOTORECEPTOR RECOVERY

In vertebrate rods and cones, photon absorption by rhodopsin or cone visual pigments triggers a phototransduction cascade that hydrolyzes cGMP, resulting in the closure of cGMP-gated cation channels on the plasma membrane that causes membrane hyperpolarization [see reviews (Pugh et al., 1997, 1999)]. To reset the resting dark-state of retinal rods (known as visual recovery), cGMP levels are replenished very quickly (Burns and Baylor, 2002) by retina-specific guanylyl cyclases (RetGCs), a membrane enzyme present in rod and cone outer segments (Dizhoor et al., 1994; Lowe et al., 1995). RetGC is a Ca^2^^+^-regulated enzyme (Koch and Stryer, 1988; Koutalos and Yau, 1996) whose activity is controlled by intracellular domains (Laura et al., 1996; Duda et al., 2005) that interact with soluble EF-hand Ca^2^^+^ sensor proteins, called guanylyl cyclase activating proteins (GCAPs: GCAP1 and GCAP2; Dizhoor et al., 1994, 1995; Gorczyca et al., 1994, 1995; Koutalos et al., 1995).

Light-induced channel closure in photoreceptor cells causes a decrease in the cytosolic free Ca^2^^+^ concentration (Gray-Keller and Detwiler, 1994), in mammals from 250 nM in the dark to 25 nM in the light (Woodruff et al., 2002). The catalytic activity of RetGC in the dark is negatively controlled by Ca^2^^+^-bound GCAPs (Dizhoor and Hurley, 1996; Dizhoor et al., 1998; Burns and Baylor, 2002), whereas the release of Ca^2^^+^ from GCAPs at low Ca^2^^+^ levels in light-activated photoreceptors causes activation of RetGC (Dizhoor et al., 1994, 1995; Gorczyca et al., 1994, 1995; Dizhoor and Hurley, 1996; Mendez et al., 2001). Light stimulation of the rod cell causes a more than 10-fold increase in cGMP production due to the activation of RetGC by GCAPs (Hodgkin and Nunn, 1988; Burns and Baylor, 2002) and is a critical step for controlling the recovery rate of a single-photon response (Pugh et al., 1999; Burns and Baylor, 2002) as well as the cone response to stronger light stimuli (Sakurai et al., 2011). In mouse rods, GCAPs have been demonstrated to have different Ca^2^^+^ sensitivities (Dizhoor et al., 1998; Hwang et al., 2003) and therefore contribute to the recovery by activating guanylyl cyclase at different steps of excitation and recovery, thus imparting proper recovery kinetics to the rod response (Mendez et al., 2001; Makino et al., 2008, 2012).

### Mg^2+^ AND Ca^2+^ BINDING TO GCAPs RECIPROCALLY CONTROL CYCLASE ACTIVATION

Guanylyl cyclase activating proteins activate RetGC at low Ca^2^^+^ levels (less than 50 nM) and only in the presence of physiological Mg^2^^+^ levels (Peshenko and Dizhoor, 2004, 2006, 2007; Dizhoor et al., 2010). This Mg^2^^+^ requirement for RetGC activation by GCAPs initially suggested that Mg^2^^+^ binding to GCAPs might be important for their activation of RetGC. Indeed, Mg^2^^+^ was shown to bind directly to at least two of the EF-hands in GCAP1 (Lim et al., 2009), and NMR studies showed that Mg^2^^+^ binding to GCAP1 at EF2 and EF3 was needed to stabilize the overall tertiary fold of the protein (Lim et al., 2009). By contrast, the Ca^2^^+^-free/Mg^2^^+^-free GCAP1 (apo-state) forms a molten globule-like structure, that contains regular secondary structure (Dell’Orco et al., 2010) but does not form a stable tertiary fold (Peshenko and Dizhoor, 2004; Lim et al., 2009). The flexible and unstructured molten-globule apo-protein could explain in part why GCAPs do not activate RetGC in the absence of Mg^2^^+^ (Dizhoor et al., 2010). Thus, Mg^2^^+^ binding to GCAP1 stablizes its protein structure in order to bind and activate RetGC (Dizhoor et al., 1994; Peshenko and Dizhoor, 2004), whereas Ca^2^^+^ binding to GCAP1 stabilizes a distinct structure important for the inhibition of RetGC (Dizhoor et al., 1998).

The four EF-hands in the GCAPs have quite distinct divalent metal binding properties that control whether GCAPs can activate or inhibit RetGC. The first EF-hand (EF1) does not bind to either Ca^2^^+^ or Mg^2^^+^ because the residue at the 3-position in the EF-hand binding loop (Cys29 in GCAP1, see **Figure 1**) is not suitable for ligating either Ca^2^^+^ or Mg^2^^+^. Ca^2^^+^ binds to GCAP1 at the other three EF-hands (EF2, EF3, and EF4) in an independent fashion (Lim et al., 2009) in contrast to the cooperative binding of two Ca^2^^+^ to recoverin (Ames et al., 1995a). The apparent dissociation constant for Ca^2^^+^ binding to GCAPs is in the submicromolar range (Lim et al., 2009; Dizhoor et al., 2010), whereas Mg^2^^+^ binds with ~1000-fold lower affinity (Gifford et al., 2007) in the sub-millimolar range (Peshenko and Dizhoor, 2004, 2007; Lim et al., 2009). These binding affinities imply that three Ca^2^^+^ bind per mole of GCAP1 in dark-adapted rod cells, which have relatively high cytosolic Ca^2^^+^ levels [Ca^2^^+^]_free_ = 250–500 nM [(Woodruff et al., 2002; Matthews and Fain, 2003) and [Mg^2^^+^] ~1 mM (Chen et al., 2003)]. Light-activation of the rod cell causes a dramatic lowering of the cytosolic Ca^2^^+^ level [Ca^2^^+^]_free_ = 5–50 nM (Gray-Keller and Detwiler, 1994; Sampath et al., 1998; Woodruff et al., 2002) while the Mg^2^^+^ level remains fixed at [Mg^2^^+^]_free_ ~1 mM (Chen et al., 2003). Therefore in light-adapted rods, GCAPs do not bind Ca^2^^+^ but instead bind to at least two Mg^2^^+^. Thus, Ca^2^^+^-free/Mg^2^^+^-bound GCAPs activate RetGC in light exposed rods (Dizhoor et al., 1994, 1995; Gorczyca et al., 1995), whereas Ca^2^^+^-bound GCAPs (with Ca^2^^+^ bound at EF2, EF3, and EF4) inhibit RetGC in dark-adapted rods (Dizhoor and Hurley, 1996; Dizhoor et al., 1998).

### CONSTITUTIVELY ACTIVE MUTANTS OF GCAP1 CAUSE RETINAL DISEASE

Various point mutations in the EF-hand motifs of GCAP1 that weaken Ca^2^^+^ binding (but do not affect Mg^2^^+^ binding) cause GCAP1 to constitutively activate RetGC in rods and cones, which is genetically linked to various retinal diseases (Jiang and Baehr, 2010). These mutations in the EF-hand motifs [Y99C (Dizhoor et al., 1998; Payne et al., 1998) and E155G (Wilkie et al., 2000, 2001)] weaken the Ca^2^^+^ binding affinity beyond the photoreceptor Ca^2^^+^ concentration and cause the Ca^2^^+^-free/Mg^2^^+^-bound GCAP1 activator state to persist even at high Ca^2^^+^ levels in dark-adapted rods, which causes persistent activation of RetGC (Sokal et al., 1998; Olshevskaya et al., 2004). The GCAP mutants that constitutively activate RetGC then cause elevated cGMP levels in photoreceptor cells that promotes apoptosis and disease (Olshevskaya et al., 2004, 2012; Woodruff et al., 2007).

Mutagenesis studies of the individual EF-hands in GCAP1 have revealed that Ca^2^^+^-binding to EF4 is critical for controlling Ca^2^^+^-dependent activation of RetGC (Peshenko and Dizhoor, 2007). Mutants of GCAP1 that weaken or abolish Ca^2^^+^ binding to EF4 but retain Ca^2^^+^ binding at EF2 and EF3 [D144N/D148G (Peshenko and Dizhoor, 2007 and E155G Wilkie et al., 2000, 2001)] are constitutively active even in the presence of high Ca^2^^+^ levels in dark-adapted photoreceptors. Furthermore, these mutants that disable Ca^2^^+^ binding to EF4 (but not EF2 and EF3) are unable to inhibit RetGC at high Ca^2^^+^ levels in the dark (Peshenko and Dizhoor, 2007). In summary, GCAP1 can activate RetGC even if Ca^2^^+^ is bound to EF2 and EF3 (but not bound to EF4). Also, Ca^2^^+^ binding to EF4 is essential for having Ca^2^^+^-induced inhibition of RetGC. Therefore, Ca^2^^+^ binding to EF4 is critical for controlling whether GCAP1 can activate or inhibit RetGC.

### GCAPs DO NOT POSSESS A Ca^2+^-MYRISTOYL SWITCH

Ca^2^^+^ binding to GCAP1 and GCAP2 does not cause ejection of the covalently attached myristoyl group (Hughes et al., 1998; Lim et al., 2009, 2013) and Ca^2^^+^ binding to GCAPs do not promote their membrane targeting (Olshevskaya et al., 1997; Hwang and Koch, 2002). This is in stark contrast to the Ca^2^^+^-induced exposure of the N-terminal myristoyl group [termed Ca^2^^+^-myristoyl switch (Zozulya and Stryer, 1992; Dizhoor et al., 1993)] that promotes membrane targeting of recoverin (Zozulya and Stryer, 1992; Dizhoor et al., 1993; Valentine et al., 2003), neurocalcin (Ladant, 1995), hippocalcin (O’Callaghan et al., 2003), VILIPs (Spilker et al., 1997, 2002), and NCS-1 (Hamasaki et al., 2004). Instead, the covalently attached myristoyl group of GCAP1 remains sequestered inside the protein hydrophobic core in both Ca^2^^+^-free and Ca^2^^+^-bound forms of GCAP1 (Lim et al., 2009, 2013). NMR studies on the myristate attached to recoverin (Ames et al., 1995b, 1997; Hughes et al., 1995), VILIP1 (Li et al., 2011), and NCS-1 (Lim et al., 2011) reveal that the covalently attached myristoyl group is buried inside these proteins only in the Ca^2^^+^-free state. Ca^2^^+^-binding causes protein conformational changes that lead to exposure of the fatty acyl chain in recoverin (Ames et al., 1997), VILIP1 (Li et al., 2011), and NCS-1 (Lim et al., 2011). By stark contrast, NMR studies on GCAP1 indicate that the covalently attached myristoyl group is buried inside both Ca^2^^+^-free and Ca^2^^+^-bound GCAP1 (Lim et al., 2009, 2013). Further evidence that GCAP1 lacks a Ca^2^^+^-myristoyl switch comes from SPR studies that show myristoylation of GCAP1 has little effect on membrane binding (Hwang and Koch, 2002). Finally, the recent atomic level structures of GCAP1 directly demonstrated that the myristoyl group is buried inside the protein in a similar environment in both the Ca^2^^+^-free activator (Lim et al., 2013) and Ca^2^^+^-bound inhibitor states (Stephen et al., 2007).

Solid-state NMR and other spectroscopic studies on GCAP2 have suggested that Ca^2^^+^-free GCAP2 might be targeted to cell membranes by a reversed Ca^2^^+^-myristoyl switch (Theisgen et al., 2010, 2011). The covalently attached fatty acyl group has been suggested to become exposed in Ca^2^^+^-free GCAP2 in the presence of lipid bilayer membranes (Theisgen et al., 2011), in contrast to having the myristoyl group sequestered inside Ca^2^^+^-bound GCAP2 (Schroder et al., 2011). However, other studies on GCAP2 indicate that the myristoyl group remains sequestered inside the protein environment in both Ca^2^^+^-free and Ca^2^^+^-bound GCAP2 (Hughes et al., 1998). Also, unmyristoylated GCAP2 activates RetGC with nearly the same potency as that of myristoylated GCAP2, and myristoylation of GCAP2 is not essential for its targeting to the membrane-bound cyclase (Olshevskaya et al., 1997; Hwang and Koch, 2002).

### Ca^2+^-INDUCED PROTEIN CONFORMATIONAL CHANGES IN GCAPs

**FIGURE 3:**
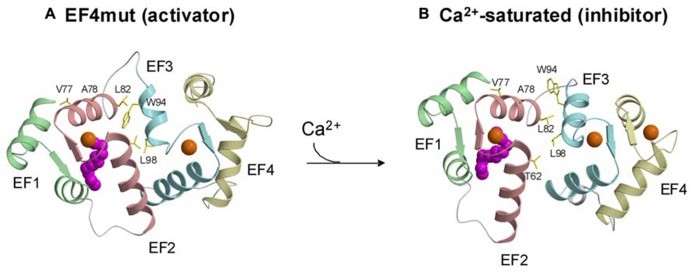
**Structures of activator (A)** vs. inhibitor forms **(B)** of GCAP1 adapted from and originally published by Lim et al. (2013).

Atomic level structures are known for Ca^2^^+^-bound forms of GCAP1 (Stephen et al., 2007) and GCAP2 (Ames et al., 1999). The four EF-hands in GCAP1 (**Figures 1** and **3**) are grouped into two globular domains: N-domain includes EF1 and EF2 (residues 18–83) and C-domain contains EF3 and EF4 (residues 88–161). Ca^2^^+^ is bound to GCAP1 at EF2, EF3 and EF4, and the structure of each Ca^2^^+^-bound EF-hand in GCAP1 (**Figure 3B**) adopts the familiar open conformation of EF-hands as seen in calmodulin and other Ca^2^^+^-bound EF-hand proteins (Ikura, 1996). Indeed, the interhelical angles for each Ca^2^^+^-bound EF-hand in GCAP1 are similar to those of recoverin (Ames et al., 1997) and NCS-1 (Bourne et al., 2001). Although the internal structure of each EF-hand in GCAP1 is similar to that of recoverin, the overall three-dimensional packing arrangement and spatial organization of the four EF-hands is very different for GCAP1 vs. recoverin. Indeed, the overall root-mean-squared deviation of main chain atoms is 3.4 Å when comparing the structures of GCAP1 and recoverin (**Figure 2**). A unique structural feature of GCAP1 is that the N-terminal α-helix (residues 5–15) upstream of EF1 and C-terminal helix (residues 175–183) downstream of EF4 are held closely together by their mutual interaction with the N-terminal myristoyl group (**Figure 2F**). Thus, the covalently attached myristoyl group in GCAP1 is sequestered within a unique environment inside the Ca^2^^+^-bound protein, which is quite different from that of recoverin as described above in **Figure 2**. The myristoyl group attached to GCAP1 makes contacts with N-terminal residues (V9, L12, and F42) and the C-terminal helix (L174, V178 and I181; **Figure 2F**). In essence, the myristoyl group serves to bridge both the N-terminal and C-terminal ends of the protein, which explains how Ca^2^^+^-induced conformational changes in the C-terminal domain (particularly in EF4) might be transmitted to a possible target binding site in EF1. A Ca^2^^+^-myristoyl tug mechanism (Peshenko et al., 2012) has been proposed to explain how Ca^2^^+^-induced conformational changes in EF4 serve to “tug” on the adjacent C-terminal helix that connects structurally to the myristoyl group and EF1. This tug mechanism helps explain how Ca^2^^+^-induced structural changes in EF4 might be relayed to the cyclase binding region in EF1 (Lim et al., 2013). The Ca^2^^+^-induced structural changes involving the C-terminal helix might also be related to Ca^2^^+^-dependent phosphorylation of S201 in GCAP2 (Peshenko et al., 2004).

The atomic level structure of Ca^2^^+^-free/Mg^2^^+^-bound activator form of GCAP1 or GCAP2 is currently not known. The main difficulty is that Ca^2^^+^-free/Mg^2^^+^-bound GCAP proteins form dimers and higher order protein oligomers that causes considerable sample heterogeneity at high protein concentrations needed for NMR or to make crystals for X-ray crystallography (Ames et al., 1999; Lim et al., 2009, 2013). Ca^2^^+^-dependent dimerization of GCAP2 has been suggested to be important for activating the cyclase (Olshevskaya et al., 1999b; Ermilov et al., 2001). Protein dimerization was also reported for GCAP1, and a GCAP1 mutant (V77E) that prevents protein dimerization also abolishes its ability to activate RetGC, suggesting that dimerization of Ca^2^^+^-free/Mg^2^^+^-bound activator state might be important for activating RetGC (Lim et al., 2013). However, protein dimerization of GCAP1 is not Ca^2^^+^ dependent and only occurs at relatively high protein concentrations above 100 μ M (Lim et al., 2013). Future studies are needed to investigate whether GCAPs might form a functional dimer upon binding to the dimeric RetGC to form a 2:2 complex (Ramamurthy et al., 2001; Peshenko et al., 2010), and whether the dimeric quaternary structure may play a regulatory role.

A GCAP1 mutant, D144N/D148G called EF4mut, (Lim et al., 2013) that binds Ca2+ at EF2 and EF3 (and does not bind Ca^2^^+^ at EF4) can activate RetGC at high Ca^2^^+^ concentrations (Peshenko and Dizhoor, 2007). Therefore, EF4mut (with Ca^2^^+^ bound at EF2 and EF3) serves as a model of the Ca^2^^+^-free/Mg^2^^+^-bound activator state. The EF4mut protein is more soluble and stable than Ca^2^^+^-free/Mg^2^^+^-bound wild type, and EF4mut exhibits NMR spectra with much higher resolution and sensitivity compared to that of Ca^2^^+^-free/Mg^2^^+^-bound wild type (Lim et al., 2009, 2013). NMR structural studies on EF4mut provide some insights for a structural model of the GCAP1 activator state (**Figure 3A**). The overall structure of EF4mut is similar to the crystal structure of Ca^2^^+^-bound GCAP1 (root mean squared deviation of main chain atoms is 1.3 Å when comparing the two structures). However, residues at the domain interface (between EF2 and EF3) are structured somewhat differently in EF4mut compared to the crystal structure of Ca^2^^+^-bound GCAP1. Many of the GCAP1 residues at the domain interface have quite broad NMR resonances, suggesting that these residues are conformationally dynamic (Lim et al., 2013). The corresponding residues in recoverin (**Figure 1**) also exhibited broad NMR resonances and ^15^N NMR relaxation dispersion studies reveal that these domain interface residues exhibit millisecond exchange kinetics (Xu et al., 2011). Ca^2^^+^-induced rearrangement of residues at the domain interface in recoverin gives rise to a 45° swiveling of the two domains (Ames et al., 1997). A structural comparison between EF4mut and Ca^2^^+^-bound GCAP1 suggests a related but much smaller Ca^2^^+^-induced structural change at the domain interface in GCAP1 (**Figure 3**). The most noteworthy Ca^2^^+^-induced structural difference in GCAP1 can be seen in the entering helix of EF3 that unravels a half turn in EF4mut, which causes a repositioning of the W94 side-chain at the domain interface. A Ca^2^^+^-induced change in the structural environment around W94 is consistent with previous tryptophan fluorescence and electron paramagnetic resonance studies of GCAP1 (Sokal et al., 2001; Peshenko and Dizhoor, 2006, 2007). We suggest that Ca^2^^+^-induced rearrangement of residues at the domain interface (V77, A78, L82, K85, and W94) plays a role in modulating Ca^2^^+^-dependent contacts with RetGC1.

### ACTIVATION MECHANISM FOR RetGC BY GCAPs

**FIGURE 4:**
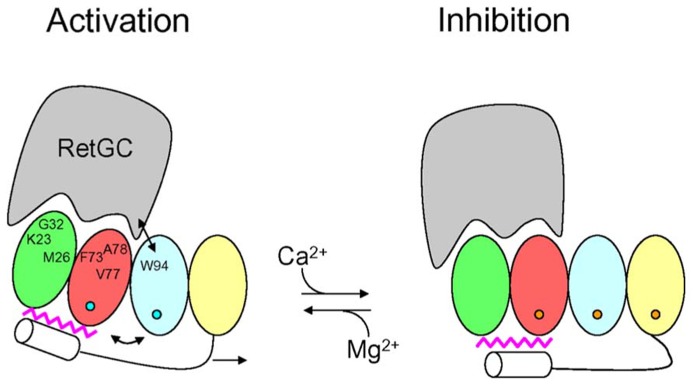
**Schematic diagram of retinal guanylyl cyclase activation by GCAP1.** RetGC is shown in gray. The four EF-hands in GCAP1 are colored coded as defined in **Figure 1**, bound Mg^2^^+^ are depicted by blue circles, bound Ca^2^^+^ are orange circles, the C-terminal helix is white, and N-terminal myristoyl group is magenta. The labeled GCAP1 residues (K23, M26, G32, F73, V77, A78, and W94) have been implicated in the RetGC binding site (Lim et al., 2013; Peshenko et al., 2014). The binding of Ca^2^^+^ at EF4 causes conformational changes that are transmitted to the RetGC binding site by a Ca^2^^+^-myristoyl tug mechanism (see arrows) modified from Peshenko et al. (2012).

The structural information for the GCAPs above provides insights into the activation mechanism of RetGC (**Figure 4**). GCAP1 residues in EF1 (Krylov et al., 1999; Olshevskaya et al., 1999a,b; Ermilov et al., 2001) and at the domain interface (Krylov et al., 1999; Lim et al., 2013; Peshenko et al., 2014) are suggested to make direct contact with RetGC (see labeled residues in **Figure 4**). In our model (**Figure 4**), Ca^2^^+^-induced conformational changes in EF4 (Lim et al., 2013) are transmitted to the cyclase binding site (see labeled residues in EF1, EF2, and EF3, **Figure 4**) by a Ca^2^^+^-myristoyl tug mechanism as described by (Peshenko et al., 2012). In the GCAP1 activator state under physiological conditions (**Figure 4**, left panel), EF2 and EF3 are bound to Mg^2^^+^ (blue circles in **Figure 4**) with EF1 and EF4 unoccupied. The Ca^2^^+^-free state of EF4 forms a loose and dynamic structure (Lim et al., 2013), which allows the adjacent C-terminal helix to reach all the way to the N-terminal myristoyl group (magenta in **Figure 4**) and thus indirectly form hydrophobic contacts with residues in EF1 and EF2. In essence, the myristoyl group forms a bridge between Ca^2^^+^-induced conformational changes in the C-terminal domain and the cyclase binding site in the N-terminal domain. In the GCAP1 activator state, residues in the cyclase binding site [see **Figure 4**, labeled residues in EF1, EF2 and EF3 (Peshenko et al., 2014)] are spatially close together and form particular contacts with RetGC that require close proximity between W94 and V77 (see arrow in **Figure 4**). In the Ca^2^^+^-bound GCAP1 inhibitor state (**Figure 4**, right panel), Ca^2^^+^ is bound to EF2, EF3, and EF4 (orange circles in **Figure 4**). Ca^2^^+^-binding to EF4 causes local conformational changes that in turn “tug” on the C-terminal helix which causes a slight reorientation of the N-terminal domain (EF1 and EF2) with respect to the C-terminal domain (EF3 and EF4). This domain swiveling causes key residues in EF3 at the domain interface (K85, W94, and K97) to move farther away from cyclase binding site residues in EF2 [F73, V77, and A78 (Peshenko et al., 2014)], which disrupts key contacts to RetGC that we suggest may be important for regulating cyclase activation.

## CONCLUSION

N-terminal myristoylation serves to remodel the structure of NCS proteins as seen for recoverin, GCAP1 and NCS-1. Each NCS protein folds differently around the attached myristoyl group, which causes each NCS protein to adopt a unique structure. Most NCS proteins possess a functional Ca^2^^+^-myristoyl switch that promotes their Ca^2^^+^-induced membrane targeting. By contrast, GCAP1 contains a sequestered myristoyl group in both its Ca^2^^+^-free and Ca^2^^+^-bound states and undergoes rather small Ca^2^^+^-induced protein conformational changes. Ca^2^^+^ binding to the fourth EF-hand in GCAP1 triggers conformational changes in the N-terminal domain via a Ca^2^^+^-myristoyl tug mechanism that controls the exposure of residues in EF1 and EF2 that we propose may serve as a target binding site for guanylyl cyclase.

## Conflict of Interest Statement

The authors declare that the research was conducted in the absence of any commercial or financial relationships that could be construed as a potential conflict of interest.

## References

[B1] Ames J. B. Ikura M. (2002). Structure and membrane-targeting mechanism of retinal Ca^2^^+^-binding proteins, recoverin and GCAP-2. *Adv. Exp. Med. Biol.* 514 333–348 10.1007/978-1-4615-0121-3_20 12596931

[B2] Ames J. B. Dizhoor A. M. Ikura M. Palczewski K. Stryer L. (1999). Three-dimensional structure of guanylyl cyclase activating protein-2, a calcium-sensitive modulator of photoreceptor guanylyl cyclases. *J. Biol. Chem.* 274 19329–19337 10.1074/jbc.274.27.19329 10383444

[B3] Ames J. B. Hendricks K. B. Strahl T. Huttner I. G. Hamasaki N. Thorner J. (2000). Structure and calcium-binding properties of Frq1, a novel calcium sensor in the yeast *Saccharomyces cerevisiae*. *Biochemistry* 39 12149–12161 10.1021/bi0012890 11015193

[B4] Ames J. B. Ishima R. Tanaka T. Gordon J. I. Stryer L. Ikura M. (1997). Molecular mechanics of calcium-myristoyl switches. *Nature* 389 198–202 10.1038/38310 9296500

[B5] Ames J. B. Lim S. Ikura M. (2012). Molecular structure and target recognition of neuronal calcium sensor proteins. *Front. Mol. Neurosci.* 5:10 10.3389/fnmol.2012.00010 PMC3275791 22363261

[B6] Ames J. B. Porumb T. Tanaka T. Ikura M. Stryer L. (1995a). Amino-terminal myristoylation induces cooperative calcium binding to recoverin. *J. Biol. Chem.* 270 4526–4533 10.1074/jbc.270.9.4526 7876221

[B7] Ames J. B. Tanaka T. Ikura M. Stryer L. (1995b). Nuclear magnetic resonance evidence for Ca(2+)-induced extrusion of the myristoyl group of recoverin. *J. Biol. Chem.* 270 30909–30913 10.1074/jbc.270.52.30909 8537345

[B8] Ames J. B. Tanaka T. Stryer L. Ikura M. (1996). Portrait of a myristoyl switch protein. *Curr. Opin. Struct. Biol.* 6 432–438 10.1016/S0959-440X(96)80106-0 8794166

[B9] An W. F. Bowlby M. R. Betty M. Cao J. Ling H. P. Mendoza G. (2000). Modulation of A-type potassium channels by a family of calcium sensors. *Nature* 403 553–556 10.1038/35000592 10676964

[B10] Augustine G. J. Santamaria F. Tanaka K. (2003). Local calcium signaling in neurons. *Neuron* 40 331–346 10.1016/S0896-6273(03)00639-1 14556712

[B11] Baehr W. Palczewski K. (2007). Guanylate cyclase-activating proteins and retina disease. *Subcell. Biochem.* 45 71–91 10.1007/978-1-4020-6191-2_4 18193635

[B12] Bernstein H. G. Baumann B. Danos P. Diekmann S. Bogerts B. Gundelfinger E. D. (1999). Regional and cellular distribution of neural visinin-like protein immunoreactivities (VILIP-1 and VILIP-3) in human brain. *J. Neurocytol.* 28 655–662 10.1023/A:1007056731551 10851344

[B13] Berridge M. J. Lipp P. Bootman M. D. (2000). The versatility and universality of calcium signalling. *Nat. Rev. Mol. Cell Biol.* 1 11–21 10.1038/35036035 11413485

[B14] Boehmerle W. Splittgerber U. Lazarus M. B. McKenzie K. M. Johnston D. G. Austin D. J. (2006). Paclitaxel induces calcium oscillations via an inositol 1,4,5-trisphosphate receptor and neuronal calcium sensor 1-dependent mechanism. *Proc. Natl. Acad. Sci. U.S.A.* 103 18356–18361 10.1073/pnas.0607240103 17114292 PMC1838755

[B15] Bondarenko V. A. Hayashi F. Usukura J. Yamazaki A. (2010). Involvement of rhodopsin and ATP in the activation of membranous guanylate cyclase in retinal photoreceptor outer segments (ROS-GC) by GC-activating proteins (GCAPs): a new model for ROS-GC activation and its link to retinal diseases. *Mol. Cell. Biochem.* 334 125–139 10.1007/s11010-009-0323-y 19941040

[B16] Bourne Y. Dannenberg J. Pollmann V. V. Marchot P. Pongs O. (2001). Immunocytochemical localization and crystal structure of human frequenin (neuronal calcium sensor1). *J. Biol. Chem.* 276 11949–11955 10.1074/jbc.M009373200 11092894

[B17] Braunewell K. H. Gundelfinger E. D. (1999). Intracellular neuronal calcium sensor proteins: a family of EF-hand calcium-binding proteins in search of a function. *Cell Tissue Res.* 295 1–12 10.1007/s004410051207 9931348

[B18] Braunewell K. H. Klein-Szanto A. J. (2009). Visinin-like proteins (VSNLs): interaction partners and emerging functions in signal transduction of a subfamily of neuronal Ca^2^^+^ -sensor proteins. *Cell Tissue Res.* 335 301–316 10.1007/s00441-008-0716-3 18989702 PMC2742949

[B19] Burgoyne R. D. Haynes L. P. (2012). Understanding the physiological roles of the neuronal calcium sensor proteins. *Mol. Brain* 5 2 10.1186/1756-6606-5-2 PMC3271974 22269068

[B20] Burgoyne R. D. O’Callaghan D. W. Hasdemir B. Haynes L. P. Tepikin A. V. (2004). Neuronal Ca^2^^+^-sensor proteins: multitalented regulators of neuronal function. *Trends Neurosci.* 27 203–209 10.1016/j.tins.2004.01.010 15046879

[B21] Burgoyne R. D. Weiss J. L. (2001). The neuronal calcium sensor family of Ca^2^^+^-binding proteins. *Biochem. J.* 353 1–12 10.1042/0264-6021:3530001 11115393 PMC1221537

[B22] Burns M. E. Baylor D. A. (2002). Activation, deactivation, and adaptation in vertebrate photoreceptor cells. *Annu. Rev. Neurosci.* 24 779–805 10.1146/annurev.neuro.24.1.779 11520918

[B23] Buxbaum J. D. Choi E. K. Luo Y. Lilliehook C. Crowley A. C. Merriam D. E. (1998). Calsenilin: a calcium-binding protein that interacts with the presenilins and regulates the levels of a presenilin fragment. *Nat. Med.* 4 1177–1181 10.1038/2673 9771752

[B24] Calvert P. D. Klenchin V. A. Bownds M. D. (1995). Rhodopsin kinase inhibition by recoverin. Function of recoverin myristoylation*. J. Biol. Chem.* 270 24127–24129 10.1074/jbc.270.27.16147 7592614

[B25] Carrion A. M. Link W. A. Ledo F. Mellstrom B. Naranjo J. R. (1999). DREAM is a Ca^2^^+^-regulated transcriptional repressor. *Nature* 398 80–84 10.1038/18044 10078534

[B26] Chen C. K. Inglese J. Lefkowitz R. J. Hurley J. B. (1995). Ca(2+)-dependent interaction of recoverin with rhodopsin kinase. *J. Biol. Chem.* 270 18060–18066 10.1074/jbc.270.30.18060 7629115

[B27] Chen C. K. Woodruff M. L. Chen F. S. Chen Y. Cilluffo M. C. Tranchina D. (2012). Modulation of mouse rod response decay by rhodopsin kinase and recoverin. *J. Neurosci.* 32 15998–16006 10.1523/JNEUROSCI.1639-12.2012 23136436 PMC3501282

[B28] Chen C. Nakatani K. Koutalos Y. (2003). Free magnesium concentration in salamander photoreceptor outer segments. *J. Physiol.* 553 125–135 10.1113/jphysiol.2003.053280 14500766 PMC2343491

[B29] Corbalan S. Gomez J. C. (2014). Signaling through C2 domains: more than one lipid target. *Biochim. Biophys. Acta* 10.1016/j.bbamem.2014.01.008 [Epub ahead of print] 24440424

[B30] Cox J. A. Durussel I. Comte M. Nef S. Nef P. Lenz S. E. (1994). Cation binding and conformational changes in VILIP and NCS-1, two neuron-specific calcium-binding proteins. *J. Biol. Chem.* 269 32807–32813 7806504

[B31] Dell’Orco D. Behnen P. Linse S. Koch K. W. (2010). Calcium binding, structural stability and guanylate cyclase activation in GCAP1 variants associated with human cone dystrophy. *Cell. Mol. Life Sci.* 67 973–984 10.1007/s00018-009-0243-8 20213926 PMC11115885

[B32] Dizhoor A. M. Boikov S. G. Olshevskaya E. V. (1998). Constitutive activation of photoreceptor guanylate cyclase by Y99C mutant of GCAP-1. Possible role in causing human autosomal dominant cone degeneration*. J. Biol. Chem.* 273 17311–17314 10.1074/jbc.273.28.17311 9651312

[B33] Dizhoor A. M. Chen C. K. Olshevskaya E. Sinelnikova V. V. Phillipov P. Hurley J. B. (1993). Role of the acylated amino terminus of recoverin in Ca(2+)-dependent membrane interaction. *Science* 259 829–832 10.1126/science.8430337 8430337

[B34] Dizhoor A. M. Ericsson L. H. Johnson R. S. Kumar S. Olshevskaya E. Zozulya S. (1992). The NH2 terminus of retinal recoverin is acylated by a small family of fatty acids. *J. Biol. Chem.* 267 16033–16036 1386601

[B35] Dizhoor A. M. Hurley J. B. (1996). Inactivation of EF-hands makes GCAP-2 (p24) a constitutive activator of photoreceptor guanylyl cyclase by preventing a Ca^2^^+^-induced “activator-to-inhibitor” transition. *J. Biol. Chem.* 271 19346–19350 10.1074/jbc.271.32.19346 8702620

[B36] Dizhoor A. M. Lowe D. G. Olsevskaya E. V. Laura R. P. Hurley J. B. (1994). The human photoreceptor membrane guanylyl cyclase, RetGC, is present in outer segments and is regulated by calcium and a soluble activator. *Neuron* 12 1345–1352 10.1016/0896-6273(94)90449-9 7912093

[B37] Dizhoor A. M. Olshevskaya E. V. Peshenko I. V. (2010). Mg^2^^+^/Ca^2^^+^ cation binding cycle of guanylyl cyclase activating proteins (GCAPs): role in regulation of photoreceptor guanylyl cyclase. *Mol. Cell. Biochem.* 334 117–124 10.1007/s11010-009-0328-6 19953307 PMC2824334

[B38] Dizhoor A. M. Olshevskaya E. V. Henzel W. J. Wong S. C. Stults J. T. Ankoudinova I. (1995). Cloning, sequencing and expression of a 24-kDa Ca^2^^+^-binding protein activating photoreceptor guanylyl cyclase. *J. Biol. Chem.* 270 25200–25206 10.1074/jbc.270.42.25200 7559656

[B39] Dizhoor A. M. Ray S. Kumar S. Niemi G. Spencer M. Rrolley D. (1991). Recoverin: a calcium sensitive activator of retinal rod guanylate cyclase. *Science* 251 915–918 10.1126/science.1672047 1672047

[B40] Duda T. Fik E. Venkataraman V. Krishnan R. Koch K. W. Sharma R. K. (2005). The calcium-sensor guanylate cyclase activating protein type 2 specific site in rod outer segment membrane guanylate cyclase type 1. *Biochemistry* 44 7336–7345 10.1021/bi050068x 15882072

[B41] Erickson M. A. Lagnado L. Zozulya S. Neubert T. A. Stryer L. Baylor D. A. (1998). The effect of recombinant recoverin on the photoresponse of truncated rod photoreceptors. *Proc. Natl. Acad. Sci. U.S.A.* 95 6474–6479 10.1073/pnas.95.11.6474 9600991 PMC27811

[B42] Ermilov A. N. Olshevskaya E. V. Dizhoor A. M. (2001). Instead of binding calcium, one of the EF-hand structures in guanylyl cyclase activating protein-2 is required for targeting photoreceptor guanylyl cyclase. *J. Biol. Chem.* 276 48143–48148 10.1074/jbc.M107539200 11584009

[B43] Frins S. Bonigk W. Muller F. Kellner R. Koch K. W. (1996). Functional characterization of a guanylyl cyclase activating protein from vertebrate rods. Cloning, heterologous expression, and localization. *J. Biol. Chem.* 271 8022–8027 10.1074/jbc.271.14.8022 8626484

[B44] Gifford J. L. Walsh M. P. Vogel H. J. (2007). Structures and metal-ion-binding properties of the Ca^2^^+^-binding helix-loop-helix EF-hand motifs. *Biochem. J.* 405 199–221 10.1042/BJ20070255 17590154

[B45] Gomez M. De Castro E. Guarin E. Sasakura H. Kuhara A. Mori I. (2001). Ca^2^^+^ signaling via the neuronal calcium sensor-1 regulates associative learning and memory in *C.* *elegans. Neuron* 30 241–248 10.1016/S0896-6273(01)00276-8 11343658

[B46] Gorczyca W. A. Polans A. S. Surgucheva I. G. Subbaraya I. Baehr W. Palczewski K. (1995). Guanylyl cyclase activating protein. A calcium-sensitive regulator of phototransduction*. J. Biol. Chem.* 270 22029–22036 10.1074/jbc.270.37.22029 7665624

[B47] Gorczyca W. A. Van Hooser J. P. Palczewski K. (1994). Nucleotide inhibitors and activators of retinal guanylyl cyclase. *Biochemistry* 33 3217–3222 10.1021/bi00177a011 7511001

[B48] Gordon J. I. Duronio R. J. Rudnick D. A. Adams S. P. Gokel G. W. (1991). Protein myristoylation. *J. Biol. Chem.* 266 8647–8650 2026581

[B49] Gray-Keller M. P. Detwiler P. B. (1994). The calcium feedback signal in the phototransduction cascade of vertebrate rods. *Neuron* 13 849–861 10.1016/0896-6273(94)90251-8 7524559

[B50] Hama H. Schnieders E. A. Thorner J. Takemoto J. Y. DeWald D. B. (1999). Direct involvement of phosphatidylinositol 4-phosphate in secretion in the yeast *Saccharomyces cerevisiae*. *J. Biol. Chem.* 274 34294–34300 10.1074/jbc.274.48.34294 10567405

[B51] Hamasaki N. Molchanova T. Takeda K. Ames J. B. (2004). Fission yeast homolog of neuronal calcium sensor-1 (Ncs1p) regulates sporulation and confers calcium tolerance. *J. Biol. Chem.* 279 12744–12754 10.1074/jbc.M311895200 14722091

[B52] Hendricks K. B. Wang B. Q. Schnieders E. A. Thorner J. (1999). Yeast homologue of neuronal frequenin is a regulator of phosphatidylinositol-4-OH kinase. *Nat. Cell Biol.* 1 234–241 10.1038/12058 10559922

[B53] Hidaka H. Okazaki K. (1993). Neurocalcin family: a novel calcium-binding protein abundant in bovine central nervous system. *Neurosci. Res.* 16 73–77 10.1016/0168-0102(93)90074-Z 8387172

[B54] Hodgkin A. L. Nunn B. J. (1988). Control of light-sensitive current in salamander rods. *J. Physiol.* 403 439–471 2473195 10.1113/jphysiol.1988.sp017258 PMC1190722

[B55] Hughes R. E. Brzovic P. S. Dizhoor A. M. Klevit R. E. Hurley J. B. (1998). Ca^2^^+^-dependent conformational changes in bovine GCAP-2. *Protein Sci.* 7 2675–2680 10.1002/pro.5560071222 9865963 PMC2143880

[B56] Hughes R. E. Brzovic P. S. Klevit R. E. Hurley J. B. (1995). Calcium-dependent solvation of the myristoyl group of recoverin. *Biochemistry* 34 11410–11416 10.1021/bi00036a013 7547868

[B57] Huttner I. G. Strahl T. Osawa M. King D. S. Ames J. B. Thorner J. (2003). Molecular interactions of yeast frequenin with Pik1. *J. Biol. Chem.* 278 4862–4874 10.1074/jbc.M207920200 12477731

[B58] Hwang J. Y. Koch K. W. (2002). Calcium- and myristoyl-dependent properties of guanylate cyclase-activating protein-1 and protein-2. *Biochemistry* 41 13021–13028 10.1021/bi026618y 12390029

[B59] Hwang J. Y. Lange C. Helten A. Hoppner-Heitmann D. Duda T. Sharma R. K. (2003). Regulatory modes of rod outer segment membrane guanylate cyclase differ in catalytic efficiency and Ca(2+)-sensitivity. *Eur. J. Biochem.* 270 3814–3821 10.1046/j.1432-1033.2003.03770.x 12950265

[B60] Ikura M. (1996). Calcium binding and conformational response in EF-hand proteins. *Trends Biochem. Sci.* 21 14–17 10.1016/0968-0004(96)80879-6 8848832

[B61] Ikura M. Ames J. B. (2006). Genetic polymorphism and protein conformational plasticity in the calmodulin superfamily: two ways to promote multifunctionality. *Proc. Natl. Acad. Sci. U.S.A.* 103 1159–1164 10.1073/pnas.0508640103 16432210 PMC1360552

[B62] Jiang L. Baehr W. (2010). GCAP1 mutations associated with autosomal dominant cone dystrophy. *Adv. Exp. Med. Biol*. 664 273–282 10.1007/978-1-4419-1399-9_31 20238026 PMC2857780

[B63] Kapp Y. Melnikov S. Shefler A. Jeromin A. Sagi R. (2003). NCS-1 and phosphatidylinositol 4-kinase regulate IgE receptor-triggered exocytosis in cultured mast cells. *J. Immunol.* 171 5320–5327 14607934 10.4049/jimmunol.171.10.5320

[B64] Kawamura S. (1993). Rhodopsin phosphorylation as a mechanism of cyclic GMP phosphodiesterase regulation by S-modulin. *Nature* 362 855–857 10.1038/362855a0 8386803

[B65] Kawamura S. Murakami M. (1991). Calcium-dependent regulation of cyclic GMP phosphodiesterase by a protein from frog retinal rods. *Nature* 349 420–423 10.1038/349420a0 1846944

[B66] Klenchin V. A. Calvert P. D. Bownds M. D. (1995). Inhibition of rhodopsin kinase by recoverin. Further evidence for a negative feedback system in phototransduction. *J. Biol. Chem.* 270 16147–16152 10.1074/jbc.270.41.24127 7608179

[B67] Kobayashi M. Takamatsu K. Saitoh S. Miura M. Noguchi T. (1992). Molecular cloning of hippocalcin, a novel calcium-binding protein of the recoverin family exclusively expressed in hippocampus [published erratum appears in *Biochem. Biophys. Res. Commun.* (1993). 196, 1017]. *Biochem. Biophys. Res. Commun*. 189 511–517 10.1016/0006-291X(92)91587-G 1280427

[B68] Kobayashi M. Takamatsu K. Saitoh S. Noguchi T. (1993). Myristoylation of hippocalcin is linked to its calcium-dependent membrane association properties. *J. Biol. Chem.* 268 18898–18904 8360179

[B69] Koch K. W. Stryer L. (1988). Highly cooperative feedback control of retinal rod guanylate cyclase by calcium ions. *Nature* 334 64–66 10.1038/334064a0 2455233

[B70] Komolov K. E. Senin I. I. Kovaleva N. A. Christoph M. P. Churumova V. A. Grigoriev I. I. (2009). Mechanism of rhodopsin kinase regulation by recoverin. *J. Neurochem.* 110 72–79 10.1111/j.1471-4159.2009.06118.x 19457073

[B71] Koutalos Y. Nakatani K. Tamura T. Yau K. W. (1995). Characterization of guanylate cyclase activity in single retinal rod outer segments. *J. Gen. Physiol.* 106 863–890 10.1085/jgp.106.5.863 8648296 PMC2229293

[B72] Koutalos Y. Yau K. W. (1996). Regulation of sensitivity in vertebrate rod photoreceptors by calcium. *Trends Neurosci.* 19 73–81 10.1016/0166-2236(96)89624-X 8820871

[B73] Krylov D. M. Niemi G. A. Dizhoor A. M. Hurley J. B. (1999). Mapping sites in guanylyl cyclase activating protein-1 required for regulation of photoreceptor membrane guanylyl cyclases. *J. Biol. Chem.* 274 10833–10839 10.1074/jbc.274.16.10833 10196159

[B74] Ladant D. (1995). Calcium and membrane binding properties of bovine neurocalcin expressed in *Escherichia coli*. *J. Biol. Chem.* 270 3179–3185 7852401

[B75] Laura R. P. Dizhoor A. M. Hurley J. B. (1996). The membrane guanylyl cyclase, retinal guanylyl cyclase-1, is activated through its intracellular domain. *J. Biol. Chem.* 271 11646–11651 10.1074/jbc.271.20.11646 8662612

[B76] Li C. Pan W. Braunewell K. H. Ames J. B. (2011). Structural analysis of Mg^2^^+^ and Ca^2^^+^ binding, myristoylation, and dimerization of the neuronal calcium sensor and visinin-like protein 1 (VILIP-1). *J. Biol. Chem.* 286 6354–6366 10.1074/jbc.M110.173724 21169352 PMC3057812

[B77] Lim S. Peshenko I. V. Dizhoor A. M. Ames J. B. (2009). Effects of Ca^2^^+^, Mg^2^^+^, and myristoylation on guanylyl cyclase activating protein 1 structure and stability. *Biochemistry* 48 850–862 10.1021/bi801897p 19143494 PMC2637916

[B78] Lim S. Peshenko I. V. Dizhoor A. M. Ames J. B. (2013). Structural insights for activation of retinal guanylate cyclase by GCAP1. *PLoS ONE *8:e81822. 10.1371/journal.pone.0081822 PMC3827477 24236217

[B79] Lim S. Strahl T. Thorner J. Ames J. B. (2011). Structure of a Ca^2^^+^- myristoyl switch protein that controls activation of a phosphatidylinositol 4-kinase in fission yeast. *J. Biol. Chem.* 286 12565–12577 10.1074/jbc.M110.208868 21288895 PMC3069458

[B80] Lowe D. G. Dizhoor A. M. Liu K. Gu Q. Spencer M. Laura R. (1995). Cloning and expression of a second photoreceptor-specific membrane retina guanylyl cyclase (RetGC), RetGC-2. *Proc. Natl. Acad. Sci. U.S.A.* 6 5535–5539 10.1073/pnas.92.12.5535 7777544 PMC41730

[B81] Makino C. L. Dodd R. L. Chen J. Burns M. E. Roca A. Simon M. I. (2004). Recoverin regulates light-dependent phosphodiesterase activity in retinal rods. *J. Gen. Physiol.* 123 729–741 10.1085/jgp.200308994 15173221 PMC2234569

[B82] Makino C. L. Peshenko I. V. Wen X. H. Olshevskaya E. V. Barrett R. Dizhoor A. M. (2008). A role for GCAP2 in regulating the photoresponse. Guanylyl cyclase activation and rod electrophysiology in GUCA1B knock-out mice. *J. Biol. Chem*. 283 29135–29143 10.1074/jbc.M804445200 PMC2570858 18723510

[B83] Makino C. L. Wen X. H. Olshevskaya E. V. Peshenko I. V. Savchenko A. B. Dizhoor A. M. (2012). A role for GCAP2 in regulating the photoresponse. Guanylyl cyclase activation and rod electrophysiology in GUCA1B knock-out mice. *PLoS ONE* 7:e47637 10.1371/journal.pone.0047637 PMC2570858 18723510

[B84] Matthews H. R. Fain G. L. (2003). The effect of light on outer segment calcium in salamander rods. *J. Physiol.* 552 763–776 10.1113/jphysiol.2003.050724 12949220 PMC2343441

[B85] McFerran B. W. Graham M. E. Burgoyne R. D. (1998). Neuronal Ca^2^^+^ sensor 1, the mammalian homologue of frequenin, is expressed in chromaffin and PC12 cells and regulates neurosecretion from dense-core granules. *J. Biol. Chem.* 273 22768–22772 10.1074/jbc.273.35.22768 9712909

[B86] Mendez A. Burns M. E. Izabela S. Dizhoor A. M. Baehr W. Palczewski K. (2001). Role of guanylate cyclase-activating proteins (GCAPs) in setting the flash sensitivity of rod photoreceptors. *Proc. Natl. Acad. Sci. U.S.A.* 98 9948–9953 10.1073/pnas.171308998 11493703 PMC55558

[B87] Moncrief N. D. Kretsinger R. H. Goodman M. (1990). Evolution of EF-hand calcium-modulated proteins. *J. Mol. Evol.* 30 522–562 10.1007/BF02101108 2115931

[B88] Nakamura T. Y. Pountney D. J. Ozaita A. Nandi S. Ueda S. Rudy B. (2001). A role for frequenin, a Ca^2^^+^-binding protein, as a regulator of Kv4 K^+^-currents. *Proc. Natl. Acad. Sci. U.S.A.* 98 12808–12813 10.1073/pnas.221168498 11606724 PMC60135

[B89] Nalefski E. A. Falke J. J. (1996). The C2 domain calcium-binding motif: structural and functional diversity. *Protein Sci.* 5 2375–2390 10.1002/pro.5560051201 8976547 PMC2143302

[B90] O’Callaghan D. W. Tepikin A. V. Burgoyne R. D. (2003). Dynamics and calcium sensitivity of the Ca^2^^+^/myristoyl switch protein hippocalcin in living cells. *J. Cell Biol.* 163 715–721 10.1083/jcb.200306042 14638856 PMC2173692

[B91] Olshevskaya E. V. Boikov S. Ermilov A. Krylov D. Hurley J. B. Dizhoor A. M. (1999a). Mapping functional domains of the guanylate cyclase regulator protein, GCAP-2. *J. Biol. Chem.* 274 10823–10832 10.1074/jbc.274.16.10823 10196158

[B92] Olshevskaya E. V. Ermilov A. N. Dizhoor A. M. (1999b). Dimerization of guanylyl cyclase-activating protein. *J. Biol. Chem.* 274 25583–25587 10.1074/jbc.274.36.25583 10464292

[B93] Olshevskaya E. V. Calvert P. D. Woodruff M. L. Peshenko I. V. Savchenko A. B. Makino C. L. (2004). The Y99C mutation in guanylyl cyclase-activating protein 1 increases intracellular Ca^2^^+^ and causes photoreceptor degeneration in transgenic mice. *J. Neurosci.* 24 6078–6085 10.1523/JNEUROSCI.0963-04.2004 15240799 PMC6729660

[B94] Olshevskaya E. V. Hughes R. E. Hurley J. B. Dizhoor A. M. (1997). Calcium binding, but not a calcium-myristoyl switch, controls the ability of guanylyl cyclase-activating protein GCAP-2 to regulate photoreceptor guanylyl cyclase. *J. Biol. Chem.* 272 14327–14333 10.1074/jbc.272.22.14327 9162068

[B95] Olshevskaya E. V. Peshenko I. V. Savchenko A. B. Dizhoor A. M. (2012). Retinal guanylyl cyclase isozyme 1 is the preferential in vivo target for constitutively active GCAP1 mutants causing congenital degeneration of photoreceptors. *J. Neurosci.* 32 7208–7217 10.1523/JNEUROSCI.0976-12.2012 22623665 PMC3368705

[B96] Palczewski K. Polans A. S. Baehr W. Ames J. B. (2000). Ca(2+)-binding proteins in the retina: structure, function, and the etiology of human visual diseases. *Bioessays* 22 337–350 10.1002/(SICI)1521-1878(200004)22:4<337::AID-BIES4>3.0.CO;2-Z 10723031

[B97] Palczewski K. Sokal I. Baehr W. (2004). Guanylate cyclase-activating proteins: structure, function, and diversity. *Biochem. Biophys. Res. Commun.* 322 1123–1130 10.1016/j.bbrc.2004.07.122 15336959

[B98] Palczewski K. Subbaraya I. Gorczyca W. A. Helekar B. S. Ruiz C. C. Ohguro H. (1994). Molecular cloning and characterization of retinal photoreceptor guanylyl cyclase-activating protein. *Neuron* 13 395–404 10.1016/0896-6273(94)90355-7 7520254

[B99] Payne A. M. Downes S. M. Bessant D. A. Taylor R. Holder G. E. Warren M. J. (1998). A mutation in guanylate cyclase activator 1A (GUCA1A) in an autosomal dominant cone dystrophy pedigree mapping to a new locus on chromosome 6p21.1. *Hum. Mol. Genet.* 7 273–277 10.1093/hmg/7.2.273 9425234

[B100] Peshenko I. V. Dizhoor A. M. (2004). Guanylyl cyclase-activating proteins (GCAPs) are Ca^2^^+^/Mg^2^^+^ sensors: implications for photoreceptor guanylyl cyclase (RetGC) regulation in mammalian photoreceptors. *J. Biol. Chem.* 279 16903–16906 10.1074/jbc.C400065200 14993224

[B101] Peshenko I. V. Dizhoor A. M. (2006). Ca^2^^+^ and Mg^2^^+^ binding properties of GCAP-1. Evidence that Mg^2^^+^-bound form is the physiological activator of photoreceptor guanylyl cyclase. *J. Biol. Chem*. 281 23830–23841 10.1074/jbc.M600257200 16793776

[B102] Peshenko I. V. Dizhoor A. M. (2007). Activation and inhibition of photoreceptor guanylyl cyclase by guanylyl cyclase activating protein 1 (GCAP-1): the functional role of Mg^2^^+^/Ca^2^^+^ exchange in EF-hand domains. *J. Biol. Chem.* 282 21645–21652 10.1074/jbc.M702368200 17545152 PMC2430010

[B103] Peshenko I. V. Olshevskaya E. V. Dizhoor A. M. (2004). Ca(2+)-dependent conformational changes in guanylyl cyclase-activating protein 2 (GCAP-2) revealed by site-specific phosphorylation and partial proteolysis. *J. Biol. Chem.* 279 50342–50349 10.1074/jbc.M408683200 15448139

[B104] Peshenko I. V. Olshevskaya E. V. Lim S. Ames J. B. Dizhoor A. M. (2012). Calcium-myristoyl Tug is a new mechanism for intramolecular tuning of calcium sensitivity and target enzyme interaction for guanylyl cyclase-activating protein 1: dynamic connection between N-fatty acyl group and EF-hand controls calcium sensitivity. *J. Biol. Chem.* 287 13972–13984 10.1074/jbc.M112.341883 22383530 PMC3340203

[B105] Peshenko I. V. Olshevskaya E. V. Lim S. Ames J. B. Dizhoor A. M. (2014). Identification of target binding site in photoreceptor guanylyl cyclase activating protein 1 (GCAP1). *J. Biol. Chem*. 289 10.1074/jbc.M113.540716 [Epub ahead of print] PMC3974984 24567338

[B106] Peshenko I. V. Olshevskaya E. V. Yao S. Ezzeldin H. H. Pittler S. J. Dizhoor A. M. (2010). Activation of retinal guanylyl cyclase RetGC1 by GCAP1: stoichiometry of binding and effect of new LCA-related mutations. *Biochemistry* 49 709–717 10.1021/bi901495y 20050595 PMC2827208

[B107] Polans A. S. Buczylko J. Crabb J. Palczewski K. (1991). A photoreceptor calcium binding protein is recognized by autoantibodies obtained from patients with cancer-associated retinopathy. *J. Cell Biol.* 112 981–989 10.1083/jcb.112.5.981 1999465 PMC2288874

[B108] Pongs O. Lindemeier J. Zhu X. R. Theil T. Engelkamp D. Krah-Jentgens I. (1993). Frequenin-a novel calcium-binding protein that modulates synaptic efficacy. *Neuron* 11 15–28 10.1016/0896-6273(93)90267-U 8101711

[B109] Pugh E. N. Duda T. Sitaramayya A. Sharma R. K. (1997). Photoreceptor guanylate cyclases: a review. *Biosci. Rep.* 17 429–473 10.1023/A:1027365520442 9419388

[B110] Pugh E. N. Nikonov S. Lamb T. D. (1999). Molecular mechanisms of vertebrate photoreceptor light adaptation. *Curr. Opin. Neurobiol.* 9 410–418 10.1016/S0959-4388(99)80062-2 10448166

[B111] Ramamurthy V. Tucker C. Wilkie S. E. Daggett V. Hunt D. M. Hurley J. B. (2001). Interactions within the coiled-coil domain of RetGC-1 guanylyl cyclase are optimized for regulation rather than for high affinity. *J. Biol. Chem.* 276 26218–26229 10.1074/jbc.M010495200 11306565

[B112] Sakurai K. Chen J. Kefalov V. J. (2011). Role of guanylyl cyclase modulation in mouse cone phototransduction. *J. Neurosci.* 31 7991–8000 10.1523/JNEUROSCI.6650-10.2011 21632921 PMC3124626

[B113] Sampath A. P. Matthews H. R. Cornwall M. C. Fain G. L. (1998). Bleached pigment produces a maintained decrease in outer segment Ca^2^^+^ in salamander rods. *J. Gen. Physiol.* 111 53–64 10.1085/jgp.111.1.53 9417134 PMC1887770

[B114] Schroder T. Lilie H. Lange C. (2011). The myristoylation of guanylate cyclase-activating protein-2 causes an increase in thermodynamic stability in the presence but not in the absence of Ca^2^^+^. *Protein Sci.* 20 1155–1165 10.1002/pro.643 21520322 PMC3149189

[B115] Semple-Rowland S. L. Gorczyca W. A. Buczylko J. Helekar B. S. Ruiz C. C. Subbaraya I. (1996). Expression of GCAP1 and GCAP2 in the retinal degeneration (rd) mutant chicken retina. *FEBS Lett.* 385 47–52 10.1016/0014-5793(96)00345-6 8641465

[B116] Sokal I. Li N. Klug C. S. Filipek S. Hubbell W. L. Baehr W. (2001). Calcium-sensitive regions of GCAP1 as observed by chemical modifications, fluorescence, and EPR spectroscopies. *J. Biol. Chem.,* 276 43361–43373 10.1074/jbc.M103614200 11524415 PMC1363678

[B117] Sokal I. Li N. Surgucheva I. Warren M. J. Payne A. M. Bhattacharya S. S. (1998). GCAP1 (Y99C) mutant is constitutively active in autosomal dominant cone dystrophy. *Mol. Cell* 2 129–133 10.1016/S1097-2765(00)80121-5 9702199

[B118] Spilker C. Dresbach T. Braunewell K. H. (2002). Reversible translocation and activity-dependent localization of the calcium-myristoyl switch protein VILIP-1 to different membrane compartments in living hippocampal neurons. *J. Neurosci.* 22 7331–7339 12196554 10.1523/JNEUROSCI.22-17-07331.2002 PMC6757958

[B119] Spilker C. Gundelfinger E. D. Braunewell K. H. (1997). Calcium- and myristoyl-dependent subcellular localization of the neuronal calcium-binding protein VILIP in transfected PC12 cells. *Neurosci. Lett.* 225 126–128 10.1016/S0304-3940(97)00201-2 9147390

[B120] Stephen R. Bereta G. Golczak M. Palczewski K. Sousa M. C. (2007). Stabilizing function for myristoyl group revealed by the crystal structure of a neuronal calcium sensor, guanylate cyclase-activating protein 1. *Structure* 15 1392–1402 10.1016/j.str.2007.09.013 17997965 PMC2556213

[B121] Stephen R. Filipek S. Palczewski K. Sousa M. C. (2008). Ca^2^^+^ -dependent regulation of phototransduction. *Photochem. Photobiol.* 84 903–910 10.1111/j.1751-1097.2008.00323.x 18346093 PMC4118144

[B122] Strahl T. Grafelmann B. Dannenberg J. Thorner J. Pongs O. (2003). Conservation of regulatory function in calcium-binding proteins: human frequenin (neuronal calcium sensor-1) associates productively with yeast phosphatidylinositol 4-kinase isoform, Pik1. *J. Biol. Chem.* 278 49589–49599 10.1074/jbc.M309017200 14512421

[B123] Strahl T. Huttner I. G. Lusin J. D. Osawa M. King D. Thorner J. (2007). Structural insights into activation of phosphatidylinositol 4-kinase (Pik1) by yeast frequenin (Frq1). *J. Biol. Chem.* 282 30949–30959 10.1074/jbc.M705499200 17720810

[B124] Strissel K. J. Lishko P. V. Trieu L. H. Kennedy M. J. Hurley J. B. Arshavsky V. Y. (2005). Recoverin undergoes light-dependent intracellular translocation in rod photoreceptors. *J. Biol. Chem.* 280 29250–29255 10.1074/jbc.M501789200 15961391

[B125] Subramanian L. Polans A. S. (2004). Cancer-related diseases of the eye: the role of calcium and calcium-binding proteins. *Biochem. Biophys. Res. Commun.* 322 1153–1165 10.1016/j.bbrc.2004.07.109 15336963

[B126] Tanaka T. Ames J. B. Harvey T. S. Stryer L. Ikura M. (1995). Sequestration of the membrane-targeting myristoyl group of recoverin in the calcium-free state. *Nature* 376 444–447 10.1038/376444a0 7630423

[B127] Theisgen S. Scheidt H. A. Magalhaes A. Bonagamba T. J. Huster D. (2010). A solid-state NMR study of the structure and dynamics of the myristoylated N-terminus of the guanylate cyclase-activating protein-2. *Biochim. Biophys. Acta* 1798 266–274 10.1016/j.bbamem.2009.06.028 19616509

[B128] Theisgen S. Thomas L. Schroder T. Lange C. Kovermann M. Balbach J. (2011). The presence of membranes or micelles induces structural changes of the myristoylated guanylate-cyclase activating protein-2. *Eur. Biophys. J.* 40 565–576 10.1007/s00249-011-0680-9 21327964

[B129] Tzingounis A. V. Kobayashi M. Takamatsu K. Nicoll R. A. (2007). Hippocalcin gates the calcium activation of the slow after hyperpolarization in hippocampal pyramidal cells. *Neuron* 53 487–493 10.1016/j.neuron.2007.01.011 17296551 PMC1832111

[B130] Valentine K. G. Mesleh M. F. Opella S. J. Ikura M. Ames J. B. (2003). Structure, topology, and dynamics of myristoylated recoverin bound to phospholipid bilayers. *Biochemistry* 42 6333–6340 10.1021/bi0206816 12767213

[B131] Walch-Solimena C. Novick P. (1999). The yeast phosphatidylinositol-4-OH kinase Pik1 regulates secretion at the Golgi. *Nat. Cell Biol.* 1 523–525 10.1038/70319 10587649

[B132] Weiss J. L. Archer D. A. Burgoyne R. D. (2000). Neuronal Ca^2^^+^ sensor-1/frequenin functions in an autocrine pathway regulating Ca^2^^+^ channels in bovine adrenal chromaffin cells. *J. Biol. Chem.* 275 40082–40087 10.1074/jbc.M008603200 11006299

[B133] Weiss J. L. Burgoyne R. D. (2002). “Neuronal calcium sensor proteins,” in *Handbook of Cell Signaling* Vol. 2 ed. Bradshaw R. (San Diego: Academic Press) 79–82

[B134] Weiss J. L. Hui H. Burgoyne R. D. (2010). Neuronal calcium sensor-1 regulation of calcium channels, secretion, and neuronal outgrowth. *Cell. Mol. Neurobiol.* 30 1283–1292 10.1007/s10571-010-9588-7 21104311 PMC11498851

[B135] Wilkie S. E. Li Y. Deery E. C. Newbold R. J. Garibaldi D. Bateman J. B. (2001). Identification and functional consequences of a new mutation (E155G) in the gene for GCAP1 that causes autosomal dominant cone dystrophy. *Am. J. Hum. Genet.* 69 471–480 10.1086/323265 11484154 PMC1235478

[B136] Wilkie S. E. Newbold R. J. Deery E. Walker C. E. Stinton I. Ramamurthy V. (2000). Functional characterization of missense mutations at codon 838 in retinal guanylate cyclase correlates with disease severity in patients with autosomal dominant cone-rod dystrophy. *Hum. Mol. Genet.* 9 3065–3073 10.1093/hmg/9.20.3065 11115851

[B137] Woodruff M. L. Olshevskaya E. V. Savchenko A. B. Peshenko I. V. Barrett R. Bush R. A. (2007). Constitutive excitation by Gly90Asp rhodopsin rescues rods from degeneration caused by elevated production of cGMP in the dark. *J. Neurosci.* 27 8805–8815 10.1523/JNEUROSCI.2751-07.2007 17699662 PMC2673730

[B138] Woodruff M. L. Sampath A. P. Mathews H. R. Krasnoperova N. V. Lem J. Fain G. L. (2002). Measurement of cytoplasmic calcium concentration in the rods of wild-type and transducin knock-out mice. *J. Physiol.* 542 843–854 10.1113/jphysiol.2001.013987 12154183 PMC2290451

[B139] Xu X. Ishima R. Ames J. B. (2011). Conformational dynamics of recoverin’s Ca^2^^+^-myristoyl switch probed by 15N NMR relaxation dispersion and chemical shift analysis. *Proteins* 79 1910–1922 10.1002/prot.23014 21465563 PMC3092842

[B140] Zozulya S. Stryer L. (1992). Calcium-myristoyl protein switch. *Proc. Natl. Acad. Sci*.* U.S.A.* 89 11569–11573 10.1073/pnas.89.23.11569 1454850 PMC50594

